# High occurrence of anti-*Toxoplasma gondii* and anti-*Neospora caninum* antibodies in dogs from the microregion of Pajeú, Sertão of the state of Pernambuco

**DOI:** 10.1007/s12639-026-01908-5

**Published:** 2026-02-05

**Authors:** Samuel Souza Silva, Renato Amorim da Silva, Jéssica de Castro Souza Carvalho, Pollyanne Rayssa Fernandes de Oliveira, Renata Pimentel Bandeira de Melo, Gílcia Aparecida de Carvalho, Rinaldo Aparecido Mota, Rafael Antonio Nascimento Ramos

**Affiliations:** 1 Laboratory of Parasitology, Federal University of the Agreste of Pernambuco, Garanhuns, Brazil; 2 Department of Veterinary Medicine, Federal Rural University of Pernambuco, Recife, Brazil; 3 Department of Veterinary Pathobiology, College of Veterinary Medicine and Biological Sciences, Texas A&M University, College Station, TX 77843 USA

**Keywords:** Toxoplasmosis, Neosporosis, Dogs, Serology, Epidemiology

## Abstract

The study aimed to determine the seroprevalence and risk factors associated with *Toxoplasma gondii* and *Neospora caninum* infections among dogs from the microregion of Pajeú, Sertão, state of Pernambuco. Blood samples (n = 247) of dogs apparently healthy were collected through puncture of cephalic vein and serum evaluated using the Immunofluorescence Antibody Test (IFAT). Risk factors were calculated using univariate analysis and logistic regression. We determined the spatial distribution of seroreactive animals through maps created using the QGIS software. The overall seroprevalence was 61.54% (152/247) and 64.78% (160/247) for *T. gondii* and *N. caninum*, respectively. Of the animals tested, 40.89% (101/247) were seroreactive for both protozoa. The presence of ectoparasites (ticks and fleas) (OR = 0.53; 0.2850–0.9931) and hunting behavior (OR = 0.45; 0.2115–0.9415) were considered risk factors for anti-*N. caninum* antibodies. Age ≥ 5 and < 10 years old (OR = 1.94; 1.0301–3.6655) and crossbreed (OR = 2.35; 1.0935–5.0652) were risk factors for seropositivity for both pathogens. Seroreactive animals were distributed heterogeneously across the microregion of Pajeú. The high occurrence of anti-*T. gondii* and anti-*N. caninum* antibodies in the dog population indicate previous exposure to these pathogens. Therefore, preventive measures should be promoted to mitigate the risk of animal and human infection.

## Introduction

*Toxoplasma gondii* and *Neospora caninum* are protozoa belonging to the phylum Apicomplexa for which, respectively, felids (e.g., *Felis catus*, *Leopardus pardalis, Puma yagouaroundi* and *P. concolor*) and canids (e.g., *Canis latrans, C. lupus familiaris*, *C. lupus dingo* and *C. lupus lupus*) are the definitive hosts (Dubey et al. 2006; Ullmann et al. 2010; Almería 2013). Both parasites are widely distributed worldwide and have a significant impact on livestock production, as abortion is observed among naturally infected animals (e.g., sheep, goats, and cattle) (Bertuzzi et al. 2022; Irehan et al. 2022). Besides its importance regarding animals, *T. gondii* can infect humans, causing illness, particularly in pregnant women and their offspring (Carvalho et al. 2021). Although *N. caninum* is not considered a zoonotic pathogen, antibodies have already been detected in humans (Lobato et al. 2006; Abo-Shehada et al. 2021) and, more recently, *N. caninum* DNA was detected in blood collected directly from the umbilical cord of a newborn in Brazil (Duarte et al. 2020).

Infection with *T. gondii* in young dogs is characterized primarily by neurological signs, including seizures, ataxia, paresis and paralysis (Garcia et al. 1999; Deiró et al. 2018). While most infected animals remain asymptomatic, susceptible dogs may exhibit any of the aforementioned clinical manifestations (Deiró et al. 2018). On the other hand, the clinical manifestations of *N. caninum* are more pronounced and commonly characterized by neuromuscular signs, such as ataxia and ascending paralysis (Robbe et al. 2016; Salama et al. 2022). Neurological syndromes and cardiac, pulmonary, reproductive, and dermatological manifestations may also be observed in congenitally infected puppies (Silva et al. 2007; Robbe et al. 2016; Souza et al. 2019).

Despite being reported worldwide, infections by these protozoa exhibit varying prevalence rates among dogs, which are typically influenced by the environment in which these animals reside, their diet, the proximity of their environment to forested areas, and the presence or absence of basic sanitation (Souza et al. 2019). For instance, seroprevalence rates for *T. gondii* have ranged from 1.8% (Japan) to 98% (Egypt) (El Behairy et al. 2013; Oi et al. 2015). For *N. caninum*, this variation ranges from 1.98% (Brazil) to 15% (Italy) (Coiro et al. 2011; Machačová et al. 2016). In Brazil, prevalence rates ranging from 5.1 to 88.5% and 0.7% to 73.85% have been observed for *T. gondii* and *N. caninum*, respectively (Santos et al. 2009; Gonçalez et al. 2010; Olbera et al. 2020; Ziemniczak et al. 2021). Previous studies conducted in the state of Pernambuco reported prevalence values ranging from 9.54 to 57.6% for *T. gondii* and 24.06% to 28.3% for *N. caninum* (Figueredo et al. 2008; Magalhães et al. 2017; Souza et al. 2019).

Serological data on the exposure of dogs to these two pathogens have indicated that the environment can potentially become contaminated by oocysts released by cats (*T. gondii*) and dogs (*N. caninum*) (Fernandes et al. 2018; Souza et al. 2019; Sevá et al. 2020). This environmental contamination is significant, as these oocysts are resistant to environmental conditions, thereby favoring their persistence even under adverse humidity and temperature conditions, such as in semi-arid regions (Ballash et al. 2019). *Toxoplasma gondii* oocysts may remain viable in the environment for up to 18 months. Although this condition has not been assessed for *N. caninum*, the close relationship between these two protozoa may indicate similar environmental resistance (Dubey et al. 2007; Shapiro et al. 2019). From an epidemiological perspective, this information is crucial and may drive effective prevention strategies. Therefore, the present study aimed to determine the seroprevalence and risk factors associated with *T. gondii* and *N. caninum* exposure among domiciled dogs in the microregion of Pajeú, Sertão, state of Pernambuco.

## Material and methods

### Study area

**Fig. 1:**
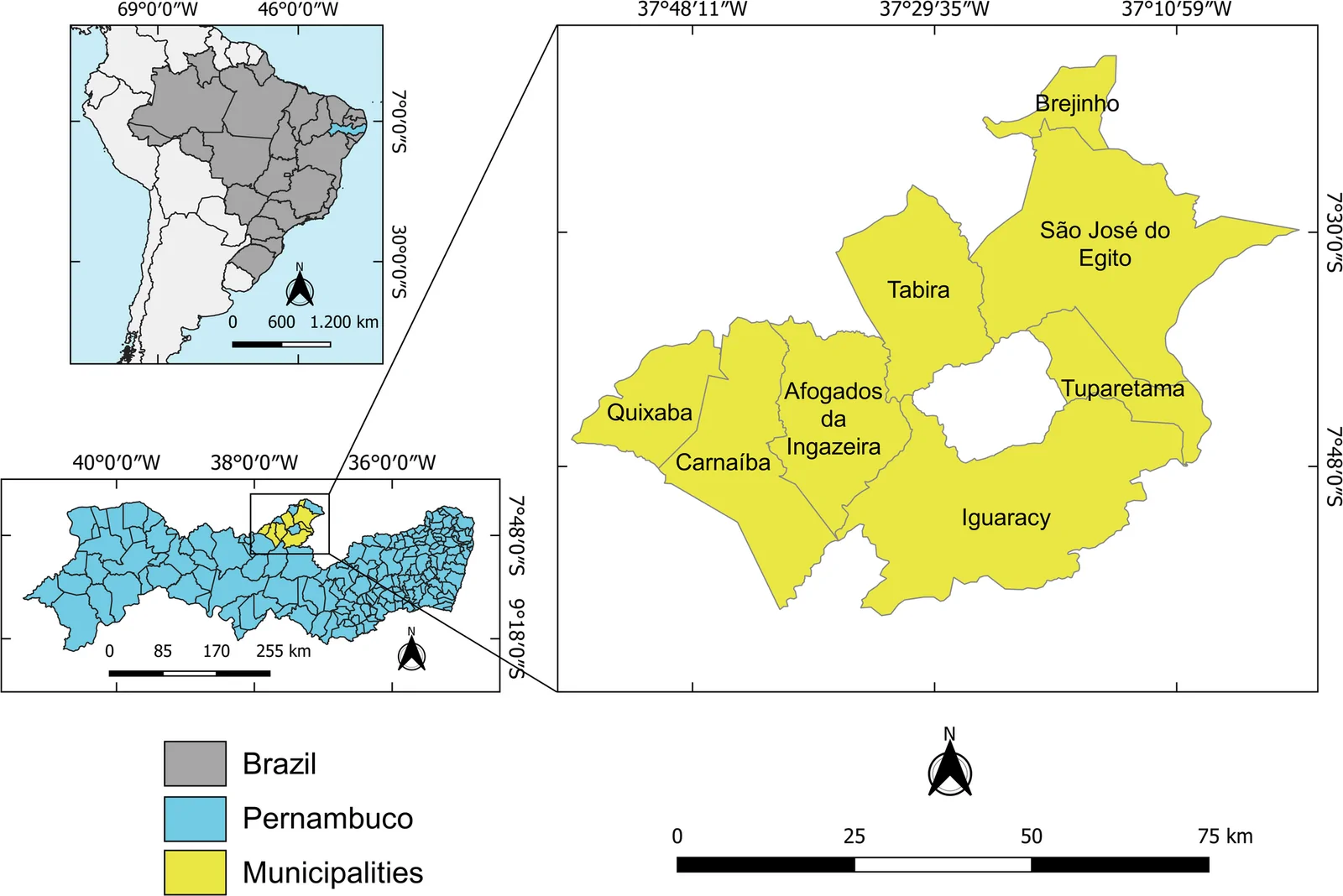
Study area: map of Brazil indicating the state of Pernambuco and municipalities of sampling

This study was carried out in eight municipalities: Afogados da Ingazeira (07°45′03'' S and 37°38′21'' W), Brejinho (07°20′58'' S and 37°17′10'' W), Carnaíba (07°48′19'' S and 37°47′38'' W), Iguaracy (07°50′07'' S and 37°30′55'' W), Quixaba (07°43′13'' S and 37°50′54'' W), São José do Egito (07°28′44'' S and 37°16′28'' W), Tabira (07°35′27'' S and 37°32′22'' W) and Tuparetama (07°36′08'' S and 37°18′41'' W). These are all located in the Pajeú microregion, Sertão of Pernambuco, northeastern Brazil (Fig. 1). This area is characterized by a hot and dry tropical climate, according to the Köppen classification (*BSh*). It lies within the caatinga biome and is formed by stretches of hyper-xerophilous forest. Its average temperature ranges from 17 to 36°C. The rainy season lasts from November to July, with an average annual precipitation ranging from 5 to 118 mm (IBGE 2021).

### Animals, sampling and serological examination

The minimum sample size determined was 247 animals. An animal/human ratio of 1:4.4, as previously determined (Canatto et al. 2012), was used for sample size calculation. The human population of the study area (35,129 inhabitants) was obtained by summing the populations of the eight municipalities. An error level of 5% and 95% confidence interval was used (Thrusfield 2004). The collection points in each municipality were randomly determined (Reis 2003).

Blood samples were collected through puncture of cephalic vein in dogs of different breeds, ages, and sexes. Serum samples were obtained by means of centrifugation at 3,500 rpm for 10 min, and stored in plastic tubes at -20°C until serological examination. An epidemiological questionnaire was applied to the dogs’ keepers at the time of this sampling in order to assess risk factors. After blood collection, a visual inspection was carried for three minutes individually in order to identify the presence of ectoparasites.

The immunofluorescence antibody test (IFAT) was performed using *T. gondii* and *N. caninum* strains ME49 and NcSp7, respectively, as antigens. The sensitization of the slides with these antigens was carried out at the Infectious Diseases Laboratory of the Federal Rural University of Pernambuco (UFRPE). The serum samples were diluted in phosphate-buffered saline (PBS), and the cut-off 1:16 and 1:50 were considered for *T. gondii* and *N. caninum*, respectively. After dilution, these samples were inserted into slide wells and incubated at 37 °C for 30 min in a humid chamber (Silva et al. 2007, 2002). After incubation, these slides were washed three times in PBS, followed by a new incubation with Anti-dog IgG conjugate (Sigma-Aldrich®, St. Louis, MO, USA) marked with fluorescein isothiocyanate diluted at 1:200. In each reaction we added positive (reactive serum of a dog from endemic area) and negative control (non-reactive serum of a dog from non-endemic area). Samples were considered positive when the entire peripheral surface of the antigen showed fluorescence at the epifluorescent microscope (OLYMPUS BX60, Bartlett, TN, USA) (Camargo 1974).

### Data analysis

The data were analyzed using descriptive statistics to obtain absolute and relative frequencies. The chi-square test was used to compare the seropositivity of dogs in urban and rural areas. Univariate analysis and logistic regression analysis were performed, considering positivity in serological tests as the dependent variable, and sex, age, breed, area, housing, hunting behavior and ectoparasites as independent variables. The Odds ratio (OR) with 95% CI were used to calculate risk factor values. The BioEstat version 5.3 (Mamirauá Institute, Pará, PA, Brazil) and EpiInfo 7.2.2.6 software (CDC, Atlanta, GA, USA) was used to analyze the chi-square test and risk factors, respectively.

### Spatial analysis

The collection points were georeferenced through the global positioning system (GPS). The QGIS software (version 3.2.10) was used, with the addition of continuous cartographic bases in shapefiles format (available in the IBGE database). A distribution map of seroreactive and non-seroreactive samples along with a Kernel density map was created to identify clusters (areas with a higher presence of seroreactive cases).

## Results

Among all dogs sampled, 175 (70.85%) were males and 72 (29.15%) were females. Overall, 152 (61.54%) were seroreactive for *T. gondii* and 160 (64.78%) for *N. caninum*. Simultaneous occurrence of antibodies was detected in 101 dogs (40.89%). The detailed results from the serological analysis, according to the municipality, are presented in Table 1.

**Table 1:** Distribution of reactive dogs for *Toxoplasma gondii* and *Neospora caninum* in the microregion of Pajeú, Sertão, state of Pernambuco

Municipality	*Toxoplasma gondii*			*Neospora caninum*			*T. gondii* and *N. caninum*			
	Urban area	Rural area	Prevalence	Urban area	Rural area	Prevalence	Urban area	Rural area	Prevalence	Animals sampled
					n/N (%)					
Afogados da Ingazeira	2/16 (12.5)	–	2 /16 (12.5)	4/16 (25)	–	4/16 (25)	9/16 (56.25)	–	9/16 (56.25)	16
Brejinho	2/12 (16.67)	–	2/12 (16.67)	1/12 (8.33)	–	1/12 (8.33)	8/12 (66.67)	-	8/12 (66.67)	12
Carnaíba	3/06 (50)	4/14 (58.57)	7/20 (58.33)	0/06	6/14 (42.86)	6/20 (30)	1/06 (46.67)	4/14 (28.57)	5/20 (25)	20
Iguaracy	0/03	-	0/03	1/03 (33.33)	-	1/03 (33.33)	0/03	-	00/03	03
Quixaba	1/07 (14.29)	31/137 (22.63)	32/144 (22.22)	3/07 (42.86)	27/137 (19.71)	30/144 (20.83)	3/07 (42.86)	62/137 (45.26)	65/144 (45.14)	144
São José do Egito	0/09	-	0/09	3/09 (33.33)	–	3/09 (33.33)	2/09 (22.22)	–	2/09 (22.22)	09
Tabira	0/01	6/33 (18.18)	6/34 (17.65)	0/01	10/33 (30.30)	10/34 (29.41)	1/01 (100)	9/33 (27.27)	10/34 (29.41)	34
Tuparetama	1/06 (16.67)	1/03 (33.33)	2/09 (22.22)	3/06 (50)	1/03 (33.33)	4/09 (44.44)	1/06 (16.67)	1/03 (33.33)	2/09 (22.22)	09
Total	9/60 (15)	42/187 (22.46)	51/247 (20.65)	15/60 (25)	44/187 (23.53)	59/247 (23.89)	25/60 (41.67)	76/187 (40.64)	101/247 (40.89)	247

n=seroreactive animals; N: sampled animals

Most of dogs seroreactive for both pathogens were from rural areas; however no statistical difference was observed between animals from rural and urban areas (χ^2^ = 1.182; *p* = 0.5537) (Table 2).

**Table 2:** Risk factors associated with seroprevalence for *Toxoplasma gondii* and *Neospora caninum* in domestic dogs from the microregion of Pajeú, Sertão, state of Pernambuco

Variables		N (%)	*T. gondii*			*N. caninum*			*T. gondii* and* N. caninum*		
			n (%)	OR (CI 95%)	*p*-value	n (%)	OR (CI 95%)	*p*-value	n (%)	OR (CI 95%)	*p*-value
Sex	Male	175 (70.85)	38 (21.71)	1.26 (0.6252–2.5346)	0.519	37 (21.14)	0.61 (0.3281–1.1316)	0.117	77 (44)	1.57 (0.8854–2.7891)	0.123
	Female	72 (29.15)	13 (18.06)	–	–	22 (30.56)	–	–	24 (33.33)	–	–
Age (years)	< 5	176 (71.26)	35 (19.89)	–	–	47 (26.70)	–	–	63 (35.80)	–	–
	≥ 5 > 10	50 (20.24)	10 (20)	1.00 (0.4591–2.2094)	0.985	10 (20)	0.68 (0.3180–1.4808)	0.337	26 (52)	1.94 (1.0301–3.6655)	0.040*
	≥ 10	21 (8.50)	06 (28.57)	1.60 (0.4950–5.1721)	0.432	02 (9.52)	0.42 (0.0839–2.1137)	0.293	12 (57.14)	1.23 (0.4407–3.4373)	0.692
Breed	Mixed	207 (83.81)	43 (20.77)	1.05 (0.4508–2.4401)	0.912	45 (21.74)	0.52 (0.2489–1.0693)	0.075	91 (43.96)	2.35 (1.0935–5.0652)	0.028*
	Purebred	40 (16.19)	08 (20)	–	–	14 (35)	–	–	10 (25)	–	–
Area	Rural	187 (75.71)	42 (22.46)	1.64 (0.7468–3.6075)	0.217	44 (23.53)	0.92 (0.4700–1.8130)	0.816	76 (40.64)	0.96 (0.5311–1.7299)	0.888
	Urban	60 (24.29)	09 (15)	–	–	15 (25)	–	–	25 (41.67)	–	–
Housing	Domicile	57 (23.08)	11 (19.30)	0.89 (0.4259–1.8881)	0.774	15 (26.32)	1.19 (0.6009–2.3371)	0.624	23 (40.35)	0.97 (0.5315–1.7752)	0.924
	Peridomicile	190 (76.92)	40 (21.05)	–	–	44 (23.16)	–	–	78 (41.05)	–	–
Hunting	Yes	69 (27.94)	18 (26.09)	1.55 (0.8039–2.9915)	0.191	10 (14.49)	0.45 (0.2115–0.9415)	0.034*	31 (44.93)	1.26 (0.7178–2.2072)	0.422
	No	178 (72.06)	33 (18.54)	–	–	49 (27.53)	–	–	70 (39.33)	–	–
Ectoparasites	Yes	103 (41.70)	21 (30.39)	0.97 (0.5205–1.8194)	0.932	18 (17.48)	0.53 (0.2850–0.9931)	0.047*	48 (46.60)	1.02 (0.6230–1.6641)	0.943
	No	144 (58.30)	30 (20.83)	–	–	41 (28.47)	–	–	53 (36.81)		–

N: total samples; n: positive samples; OR: odds ratio; CI: confidence interval. *p < 0.05: significant association

Among all variables assessed, the presence of ectoparasites (ticks and fleas) (OR = 0.53; *p* = 0.047) and hunting behavior (OR = 0.45; *p* = 0.034) were identified as risk factors for anti-*N. caninum* . Age from 5 to 10 years old (OR = 1.94; *p* = 0.040) and crossbreed (OR = 2.53; *p* = 0.028) were risk factors when seroreactivity for both pathogens was assessed. No risk factors were observed for animals that presented antibodies only for *T. gondii* (Table 2).

**Fig. 2:**
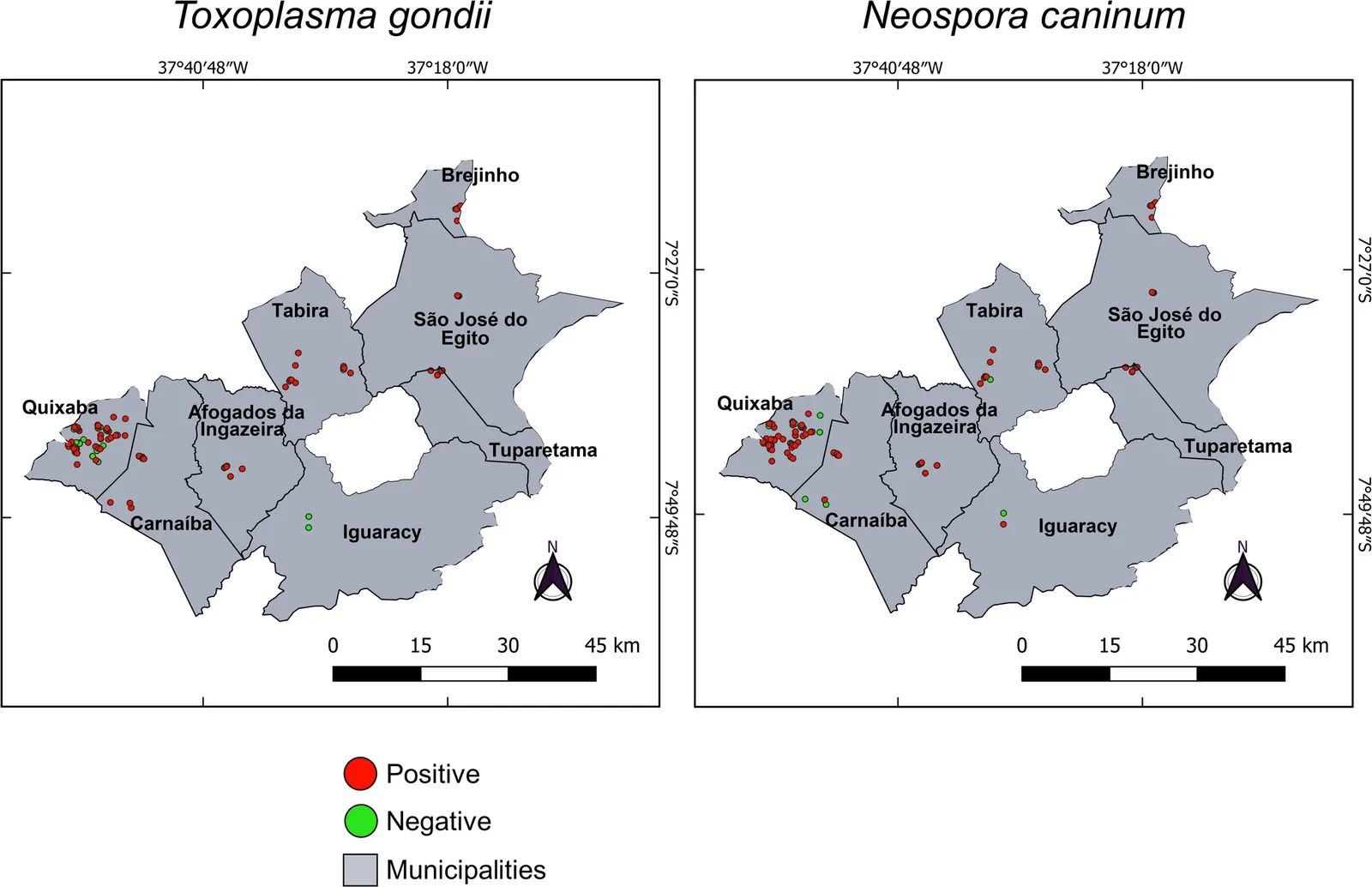
Spatial distribution of animals seropositive and seronegative for *Toxoplasma gondii* and *Neospora caninum*

Spatial analysis revealed a heterogeneous distribution of dogs that were seroreactive for *T. gondii* and *N. caninum* (Fig. 2).

**Fig. 3:**
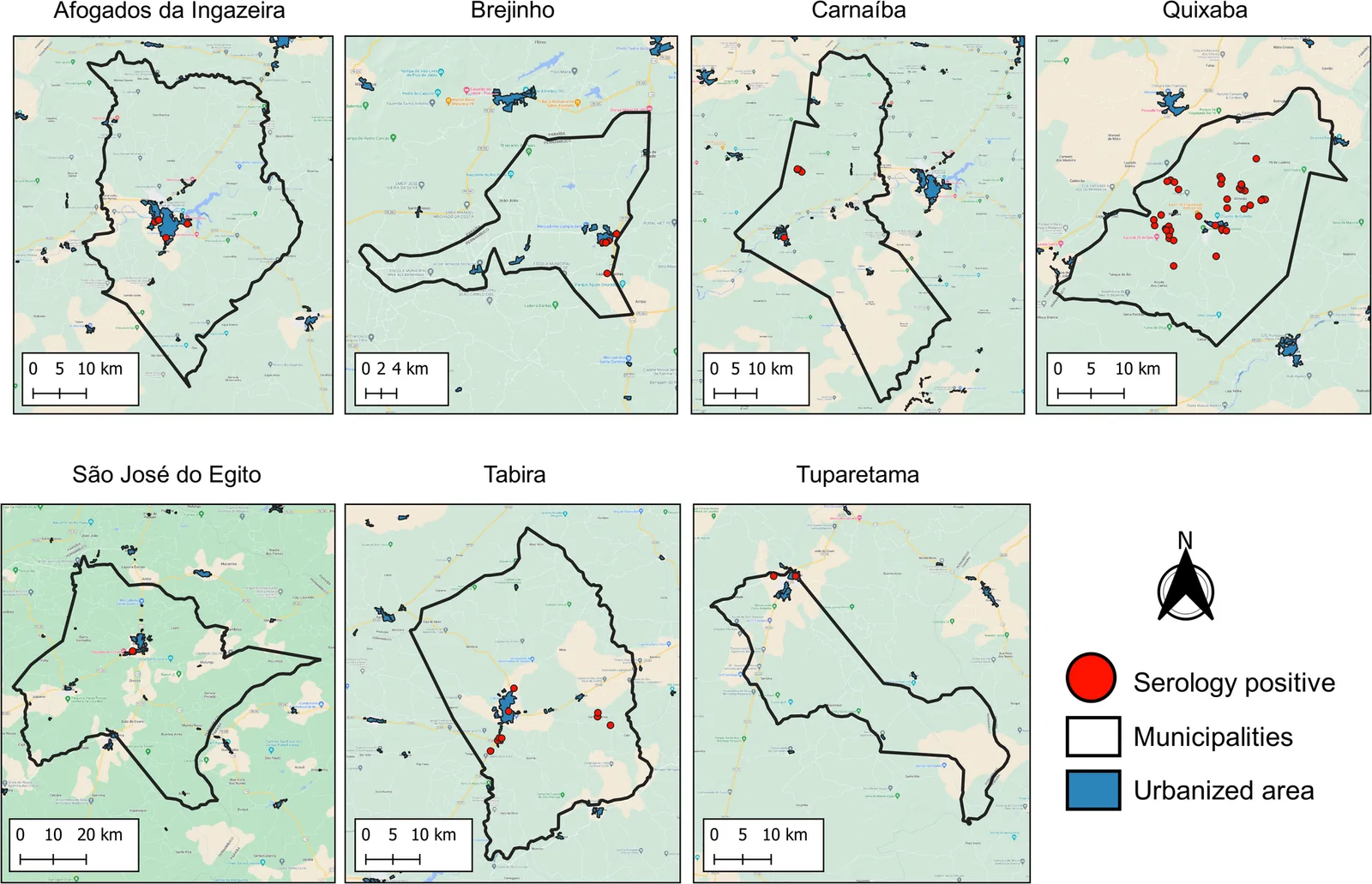
Spatial distribution of animals seropositive for *Toxoplasma gondii* and *Neospora caninum*, with delimitation of urban and rural limits

Seroreactive animals were observed in both urban and rural areas of Brejinho, Carnaíba, Quixaba, Tabira and Tuparetama, while in Afogados da Ingazeira and São José do Egito, the seroreactivity was detected exclusively in urban areas (Fig. 3). No seroreactive animals were observed in the municipality of Iguaracy.

**Fig. 4:**
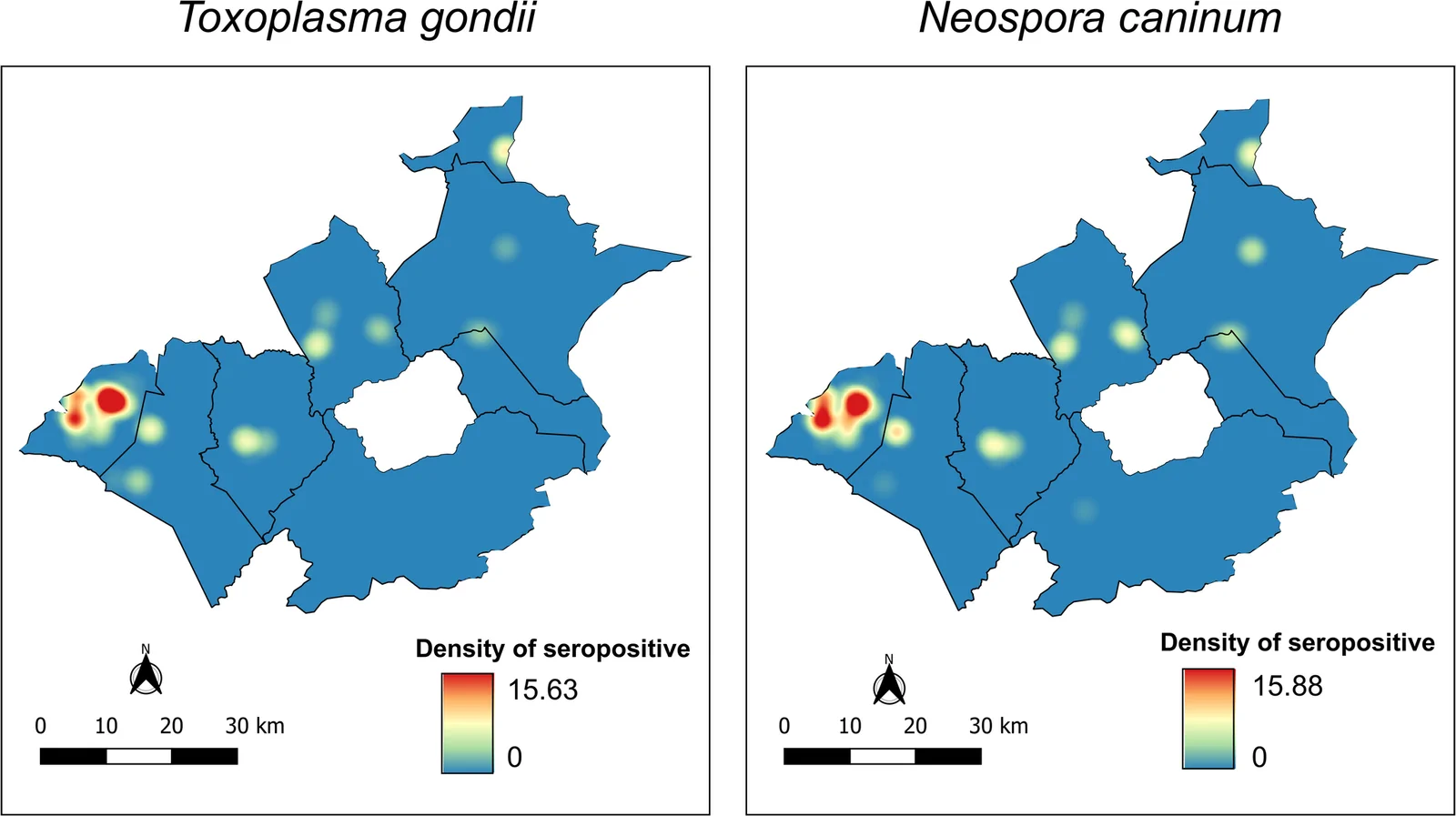
Kernel density map indicating areas with clusters of animals seroreactive for *Toxoplasma gondii* and *Neospora caninum*

A kernel density map revealed the presence of clusters of seroreactive animals. For *T. gondii*, a marked cluster was observed in the municipality of Quixaba (rural and urban areas) and with less intensity in all other municipalities. For *N. caninum*, a higher-intensity cluster was observed in the municipality of Quixaba (rural and urban areas). Despite being less intense, clusters were also observed in all other municipalities (Fig. 4).

## Discussion

This study demonstrated a high occurrence of antibodies anti-*T. gondii* and *N. caninum* among domiciled dogs living in a semi-arid region. The overall prevalence for *T. gondii* (61.57%) and *N. caninum* (64.78%) was higher than in several studies conducted previously in the northeastern region of Brazil, in which the seroprevalence ranged from 9.6 to 15.6% for *T. gondii* in the municipality of João Pessoa and from 1.6 to 7.5% to *N. caninum* in the municipality of Campina Grande (Dantas et al. 2014; Brasil et al. 2018). It is crucial to emphasize that the lower prevalence rates detected in those previous studies may have been related to the type of sample used. All of those dogs were enrolled in veterinary clinics, which suggests that they received veterinary care such as deworming, vaccination, and regular check-ups, which may have had an impact on the final outcome of the serosurvey (Brasil et al. 2018). Seroreactive animals predominated in rural areas, where the likelihood of exposure to other intermediate hosts is higher than for dogs living in urban areas (Raimundo et al. 2015).

Hunting behavior was considered to be a risk factor associated with the presence of anti-*N. caninum* antibodies. Most likely, this occurred due to the exposure of these dogs to raw meat offered by hunters after the evisceration of hunted animals. Despite a lack of local studies demonstrating that hunted animals in this region harbor *N. caninum*, this possibility has been observed in other regions in boars, wild rabbits, and hares (Machačová et al. 2016). Interestingly, the presence of ectoparasites was also a risk factor. *Neospora caninum* is not transmitted by vectors; however, vector-borne pathogens (e.g., *Anaplasma platys*, *Ehrlichia canis*, *Babesia canis*, *Hepatozoon canis* and *Mycoplasma haemocanis*) may cause immunodepression in dogs, thereby favoring infection by other pathogens. Only exposure to *T. gondii* and *N. caninum* was assessed in the present study, but we cannot rule out the possibility of co-infections with vector-borne pathogens facilitating *N. caninum* infection (Santos et al. 2009; Ramos et al. 2010; Coelho et al. 2013).

Although crossbreeding was considered a risk factor for both *T. gondii* and *N. caninum* exposure, the characteristics of the dog population may have influenced these results. The number of crossbred dogs sampled was approximately five times higher than the number of purebred dogs. Another risk factor related to exposure to both agents was age ≥ 5 and < 10 years. Several studies have shown that the probability of exposure to *T. gondii* and *N. caninum* increases with age, a relationship that is likely due to longer exposure times (Azevedo et al. 2005; Collantes-Fernandéz et al. 2008; Covre et al. 2022).

The spatial analysis showed a broader distribution of seroreactive to both pathogens. The raster layer formed in the kernel map was similar for both pathogens, thus indicating that the epidemiological characteristics of the two agents may be strongly related. Although the study area was located in a semi-arid region, the high rates of seroreactivity indicated that this locality presents favorable conditions for the circulation of *T. gondii* and *N. caninum* among animal and, potentially, human populations.

This study was the first carried out in the microregion of Pajeú, within the mesoregion of the Sertão of Pernambuco. It detected anti-*T. gondii* and anti-*N. caninum* antibodies in dogs and showed that the presence of ectoparasites and hunting behavior are risk factors for the presence of anti-*N. caninum*; and that age ≥ 5 and < 10 and mixed breed are risk factors for the presence of antibodies to both agents. Preventive measures (e.g., health education, not feeding raw meat to dogs, and avoiding the availability of offal and raw meat to animals during hunting) need to be promoted to improve dog owners' awareness about the risk factors associated with animal infection.

## References

[CR1] Abo-Shehada MN, Khalil R, Abu-Halaweh M, Sweis K, Amr Z, Billeh L (2021) Seroprevalences of *Toxoplasma gondii* and *Neospora caninum* infections in Jordanian women who had a recent spontaneous abortion. Rev Bras Parasitol Vet 30:e 008821. 10.1590/S1984-29612021076 34586175

[CR2] Almería S (2013) *Neospora caninum* and wildlife. Int Sch Res Notices 2013:1–24. 10.5402/2013/947347

[CR3] Azevedo SS, Batista CSA, Vasconcellos SA, Aguiar DM, Ragozo AMA, Rodrigues AAR, Alves CJ, Gennari SM (2005) Seroepidemiology of *Toxoplasma gondii* and *Neospora caninum* in dogs from the state of Paraíba, Northeast region of Brazil. Res Vet Sci 79:51–56. 10.1016/j.rvsc.2004.10.001 15894024

[CR4] Ballash GA, Jenkins MC, Kwok OCH, Dubey JP, Shoben AB, Robison TL, Kraft T, Shaffer EE, Dennis PM (2019) Effect of urbanization on *Neospora caninum* seroprevalence in white-tailed deer (*Odocoileus virginianus*). EcoHealth 16:109–115. 10.1007/s10393-018-1390-x 30627981

[CR5] Bertuzzi CA, Weschenfelder EA, Webber DA, Martins TA, Nino BSL, Frigotto TA, Garcia JL, Zulpo DL (2022) Soroprevalência de *Toxoplasma gondii*, *Neospora caninum* e *Brucella abortus* em ovinos de Toledo, Paraná, Brasil. Semina: Ciênc Agrár 43:2657–2670. 10.5433/1679-0359.2022v43n6p2657

[CR6] Brasil AWL, Parentoni RN, Silva JG, Santos CSAB, Mota RA, Azevedo SS (2018) Risk factors and anti-*Toxoplasma gondii* and *Neospora caninum* antibody occurrence in dogs in João Pessoa, Paraíba state, Northeastern Brazil. Rev Bras Parasitol Vet 27:242–247. 10.1590/S1984-29612018006 29846448

[CR7] Camargo ME (1974) Introdução às técnicas de imunofluorescência. Rev Bras Patol Clin 10:87–107

[CR8] Canatto BD, Silva EA, Bernardi F, Mendes MCNC, Paranhos NT, Dias RA (2012) Caracterização demográfica das populações de cães e gatos supervisionados do município de São Paulo. Arq Bras Med Vet Zootec 64:1515–1523. 10.1590/S0102-09352012000600017

[CR9] Carvalho MC, Ribeiro-Andrade M, Melo RPB, Guedes DM, Junior JWP, Cavalcanti EFTSF, Magalhães FJR, Mota RA (2021) Cross-sectional survey for *Toxoplasma gondii infection* in humans in Fernando de Noronha island, Brazil. Rev Bras Parasitol Vet 30:e005121. 10.1590/S1984-29612021062 34259739

[CR10] Coelho WMD, Coelho JA, Teixeira WFP, Coelho NMD, Oliveira GP, Lopes WDZ, Cruz BC, Maciel WG, Soares VE, Bresciani KDS (2013) Detection of co-infections by *Leishmania* (*L.*) *chagasi*, *Trypanosoma evansi*, *Toxoplasma gondii* and *Neospora caninum* in dogs. Ars Vet 29:169–174. 10.15361/2175-0106.2013v29n3p169-174

[CR11] Coiro CJ, Langoni H, Silva RC, Ullmann LS (2011) Fatores de risco para leptospirose, leishmaniose, neosporose e toxoplasmose em cães domiciliados e peridomíciliados em Botucatu-SP. Vet Zootec 18:393–407

[CR12] Collantes-Fernández E, Gómez-Bautista M, Miró G, Ávarez-García G, Pereira-Bueno J, Frisuelos C, Ortega-Mora LM (2008) Seroprevalence and risk factors associated with *Neospora caninum* infection in different dog populations in Spain. Vet Parasitol 152:148–151. 10.1016/j.vetpar.2007.12.005 18241992

[CR13] Covre KC, Farias PCGF, Barros RAM, Amorim VG, Salaroli LB, Lopes REN, Vitor RWA, Fux B (2022) Frequency of anti-*Toxoplasma gondii* antibodies in dogs and cats from the metropolitan region of Vitória, Espírito Santo, Brazil. Arch Vet Sci 27:1–7. 10.1016/j.vetpar.2007.12.005

[CR14] Dantas SBA, Fernandes ARF, Neto OLS, Mota RA, Alves CJ, Azevedo SS (2014) Risk factors associated with the occurrence of antibodies against *Toxoplasma gondii* and *Neospora caninum* in domiciled dogs in Northeastern Brazil. Semina Cienc Agrar 35:875–881. 10.5433/1679-0359.2018v39n1p199

[CR15] Deiró AG, Montargil SMA, Carvalho FS, Munhoz AD, Albuquerque GR (2018) Antibody occurrence of anti-*Toxoplasma gondii*, *Leishmania* sp. and *Ehrlichia canis* in dogs in Bahia State. Semina Cienc Agrar 39:199–210. 10.5433/1679-0359.2018v39n1p199

[CR16] Duarte PO, Oshiro LM, Zimmermann NP, Csordas BG, Dourado DM, Barros JC, Andreotti R (2020) Serological and molecular detection of *Neospora caninum* and *Toxoplasma gondii* in human umbilical cord blood and placental tissue samples. Sci Rep 10:9043. 10.1038/s41598-020-65991-1 32493968 PMC7271125

[CR17] Dubey JP, Chapman JL, Rosenthal BM, Mense M, Schueler RL (2006) Clinical *Sarcocystis neurona*, *Sarcocystis canis*, *Toxoplasma gondii*, and *Neospora caninum* infections in dogs. Vet Parasitol 137:36–49. 10.1016/j.vetpar.2005.12.017 16458431

[CR18] Dubey JP, Schares G, Mora O (2007) Epidemiology and control of neosporosis and *Neospora**caninum*. Clin Microbiol Rev 20:323–367. 10.1128/cmr.00031-06 17428888 PMC1865591

[CR19] El Behairy AM, Choudhary S, Ferreira LR, Kwok OCH, Hilali M, Su C, Dubey JP (2013) Genetic characterization of viable *Toxoplasma gondii* isolates from stray dogs from Giza, Egypt. Vet Parasitol 193:25–29. 10.1016/j.vetpar.2012.12.007 23333072

[CR20] Fernandes ARF, Costa DF, Andrade MR, Bezerra CS, Mota RA, Aves CJ, Langoni H, Azevedo SS (2018) Soropositividade e fatores de risco para leptospirose, toxoplasmose e neosporose na população canina do Estado da Paraíba. Pesq Vet Bras 38:957–966. 10.1590/1678-5150-PVB-5137

[CR21] Figueredo LA, Dantas-Torres F, Faria EB, Gondim LFP, Simões-Matos L, Brandão-Filho SP, Mota RA (2008) Occurrence of antibodies to *Neospora caninum* and *Toxoplasma gondii* in dogs from Pernambuco, Northeast Brazil. Vet Parasitol 157:9–13. 10.1016/j.vetpar.2008.07.009 18723288

[CR22] Garcia JL, Navarro IT, Ogawa L, Oliveira RC (1999) Soroepidemiologia da toxoplasmose em gatos e cães de propriedades rurais do município de Jaguapitã, estado do Paraná, Brasil. Cienc Rur 29:99–104. 10.1590/S0103-84781999000100018

[CR23] Gonçalez CC, Paes AC, Langoni H, Silva RC, Greca H, Camossi LG, Guimarães FF, Ullmann LS (2010) Anticorpos para *Leptospira* spp., *Toxoplasma gondii* e *Neospora caninum* em cães errantes albergados em canil privado. Arq Bras Med Vet Zootec 62:1011–1014. 10.1590/S0102-09352010000400037

[CR24] IBGE (2021) Cidades https://cidades.ibge.gov.br/. Acessed 13 June 2023

[CR25] Irehan B, Sonmez A, Atalay MM, Ekinci AI, Celik F, Durmus N, Ciftci AT, Simsek S (2022) Investigation of *Toxoplasma gondii*, *Neospora caninum* and *Tritrichomonas foetus* in abortions of cattle, sheep and goats in Turkey: analysis by real-time PCR, conventional PCR and histopathological methods. Comp Immunol Microbiol Infect Dis 89:101867. 10.1016/j.cimid.2022.101867 36087449

[CR26] Lobato J, Silva DAO, Mineo TWP, Amaral JDHF, Segundo GRS, Costa-Cruz JM, Ferreira MS, Borges AS, Mineo JR (2006) Detection of immunoglobulin G antibodies to *Neospora caninum* in humans: high seropositivity rates in patients who are infected by human immunodeficiency virus or have neurological disorders. Clin Vaccine Immunol 13:84–89. 10.1128/CVI.13.1.84-89.2006 16426004 PMC1356624

[CR27] Machačová T, Bártová E, Sedlák K, Slezáková R, Budíková M, Piantedosi D, Veneziano V (2016) Seroprevalence and risk factors of infections with *Neospora caninum* and *Toxoplasma gondii* in hunting dogs from Campania region, southern Italy. Folia Parasitol 63:1–5. 10.14411/fp.2016.012 27189127

[CR28] Magalhães FJR, Ribeiro-Andrade M, Souza FM, Filho CDFL, Biondo AW, Vidotto O, Navarro IT, Mota RA (2017) Seroprevalence and spatial distribution of *Toxoplasma gondii* infection in cats, dogs, pigs and equines of the Fernando de Noronha Island, Brazil. Parasitol Int 66:43–46. 10.1016/j.parint.2016.11.014 27894907

[CR29] Oi M, Yoshikawa S, Maruyama S, Nogami S (2015) Comparison of *Toxoplasma gondii* seroprevalence in shelter cats and dogs during 1999–2001 and 2009–2011 in Tokyo, Japan. PLoS ONE 10:e0135956. 10.1371/journal.pone.0135956 26284780 PMC4540423

[CR30] Olbera AVG, Fornazari F, Babboni SD, Rossi RS, Sevá AP, Latosinski GS, Silva MARX, Modolo JR, Langoni H (2020) Cumulative incidence and spatial distribution of dogs exposed to *Toxoplasma gondii*. Rev Bras Parasitol Vet 29:e000820. 10.1590/S1984-29612020025 32490893

[CR31] Raimundo JM, Guimarães A, Moraes LMB, Santos LA, Nepomuceno LL, Barbosa SM, Pires MS, Santos HA, Massard CL, Machado RZ, Baldani CD (2015) *Toxoplasma gondii* and *Neospora caninum* in dogs from the state of Tocantins: serology and associated factors. Rev Bras Parasitol Vet 24:475–481. 10.1590/S1984-29612015068 26689184

[CR32] Ramos R, Ramos C, Araújo F, Oliveira R, Souza I, Pimentel D, Galindo M, Santana M, Rosas E, Faustino M, Alves L (2010) Molecular survey and genetic characterization of tick-borne pathogens in dogs in metropolitan Recife (northeastern Brazil). Parasitol Res 107:1115–1120. 10.1007/s00436-010-1979-7 20680344

[CR33] Reis JC (2003) Estatística aplicada à pesquisa em ciência veterinária. Recife, Copyright.

[CR34] Robbe D, Passarelli A, Gloria A, Cesari AD, Capelli G, Iorio R, Tavares D (2016) *Neospora caninum* seropositivity and reproductive risk factors in dogs. Exp Parasitol 164:31–35. 10.1016/j.exppara.2016.02.003 26873272

[CR35] Salama DB, Fereig RM, Abdelbaky HH, Shahat MS, Arafa WM, Aboelhadid SM, Mohamed AEA, Metwally S, Abas O, Suo X, Gupta N, Frey CF (2022) *Toxoplasma gondii* and *Neospora caninum* antibodies in dogs and cats from Egypt and risk factor analysis. Pathogens 11:1464. 10.3390/pathogens11121464 36558798 PMC9785966

[CR36] Santos F, Coppede JS, Pereira ALA, Oliveira LP, Roberto PG, Benedetti RBR, Zucoloto LB, Lucas F, Sobreira L, Martins M (2009) Molecular evaluation of the incidence of *Ehrlichia canis*, *Anaplasma platys* and *Babesia* spp. in dogs from Ribeirão Preto, Brazil. Vet J 179:145–148. 10.1016/j.tvjl.2007.08.017 17920967

[CR37] Sevá AP, Chiebao DP, Brandão APD, Godoy SN, Jimenez-Villegas T, Pena HFJ, Ferreira F (2020) Seroprevalence and incidence of *Toxoplasma gondii* and *Neospora caninum* infection in naturally exposed domestic dogs from a rural area of São Paulo state, Brazil. Rev Bras Parasitol Vet 29:e008820. 10.1590/S1984-29612020053 33027425

[CR38] Shapiro K, Bahia-Oliveira L, Dixon B, Dumètre A, Wit LA, VanWormer E, Villena I (2019) Environmental transmission of *Toxoplasma gondii*: oocysts in water, soil and food. Food Water Parasitol 15:e00049. 10.1016/j.fawpar.2019.e00049 PMC7033973 32095620

[CR39] Silva NM, Lourenço EV, Silva DAO, Mineo JR (2002) Optimization of cut-off titres in *Toxoplasma gondii* specific ELISA and IFAT in dog sera using immunoreactivity to SAG-1 antigen as a molecular marker of infection. Vet J 163:94–98. 10.1053/tvjl.2001.0629 11749142

[CR40] Silva DAO, Lobato J, Mineo TWP, Mineo JR (2007) Evaluation of serological tests for the diagnosis of *Neospora caninum* infection in dogs: optimization of cut off titers and inhibition studies of cross-reactivity with *Toxoplasma gondii*. Vet Parasitol 143:234–244. 10.1016/j.vetpar.2006.08.028 16973287

[CR41] Souza IB, Fernandes PR, Silva TRM, Santos CVB, Silva NMM, Filho CRCU, Carvalho GA, Alves LC, Mota RA, Ramos RAN (2019) Seroprevalence of *Neospora caninum* and *Toxoplasma gondii* in dogs from an urban area of Northeastern Brazil: a spatial approach. Rev Soc Bras Med Trop 52:e20180440. 10.1590/0037-8682-0440-2018 30994810

[CR42] Thrusfield MV (2004) Epidemiologia veterinária. São Paulo, Roca.

[CR43] Ullmann LS, Silva RC, Moraes W, Cubas ZS, Santos LC, Hoffmann JL, Moreira N, Guimaraes AMS, Montaño P, Langoni H, Biondo AW (2010) Serological survey of *Toxoplasma gondii* in captive Neotropical felids from Southern Brazil. Vet Parasitol 172:144–146. 10.1016/j.vetpar.2010.04.013 20472340

[CR44] Ziemniczak HM, Maia MO, Maia MO, Ferreira E, Vieira NT, Saturnino KC, Bresciani KDS, Gomes AAD, Pacheco RC, Santos-Doni TR (2021) High seroprevalence of *Toxoplasma gondii* and *Neospora* spp. in stray dogs from Rolim de Moura, Rondônia state, Western Brazilian Amazon. Semina Cienc Agrar 42:3535–3542. 10.5433/1679-0359.2021v42n6p3535

